# PΨFinder: a practical tool for the identification and visualization of novel pseudogenes in DNA sequencing data

**DOI:** 10.1186/s12859-022-04583-4

**Published:** 2022-02-03

**Authors:** Sanna Abrahamsson, Frida Eiengård, Anna Rohlin, Marcela Dávila López

**Affiliations:** 1 Bioinformatics Core Facility, Sahlgrenska Academy, University of Gothenburg, Box 115, 405 30 Gothenburg, Sweden; 2 Department of Laboratory Medicine, Institute of Biomedicine, Sahlgrenska Academy, University of Gothenburg, Gothenburg, Sweden; 3 Unit of Genetic Analysis and Bioinformatics, Department of Clinical Genetics and Genomics, Sahlgrenska University Hospital, Gothenburg, Sweden

**Keywords:** Processed pseudogenes, DNA sequencing, Colorectal cancer, *SMAD4*, *CBX3*, *C15ORF57*, *SCAI*

## Abstract

**Background:**

Processed pseudogenes (PΨgs) are disabled gene copies that are transcribed and may affect expression of paralogous genes. Moreover, their insertion in the genome can disrupt the structure or the regulatory region of a gene, affecting its expression level. These events have been identified as occurring mutations during cancer development, thus being able to identify PΨgs and their location will improve their impact on diagnostic testing, not only in cancer but also in inherited disorders.

**Results:**

We have implemented PΨFinder (P-psy-finder), a tool that identifies PΨgs, annotates known ones and predicts their insertion site(s) in the genome. The tool screens alignment files and provides user-friendly summary reports and visualizations. To demonstrate its applicability, we scanned 218 DNA samples from patients screened for hereditary colorectal cancer. We detected 423 PΨgs distributed in 96% of the samples, comprising 7 different parent genes. Among these, we confirmed the well-known insertion site of the *SMAD4-*PΨg within the last intron of the *SCAI* gene in one sample. While for the ubiquitous *CBX3-*PΨg, present in 82.6% of the samples, we found it reversed inserted in the second intron of the *C15ORF57* gene.

**Conclusions:**

PΨFinder is a tool that can automatically identify novel PΨgs from DNA sequencing data and determine their location in the genome with high sensitivity (95.92%). It generates high quality figures and tables that facilitate the interpretation of the results and can guide the experimental validation. PΨFinder is a complementary analysis to any mutational screening in the identification of disease-causing mutations within cancer and other diseases.

**Supplementary Information:**

The online version contains supplementary material available at 10.1186/s12859-022-04583-4.

## Background

Pseudogenes (Ψgs) are abundant and ubiquitous protein-coding gene copies that are originally derived from functional genes [1]. These have been widely known as “junk” DNA for many years [2]. However, nowadays there is evidence of a handful of functional Ψgs [3–7]. For instance, some can interfere with their parental counterparts in tumorigenesis by retaining or gaining protein coding properties [7–9]. Cheetham et al. [10] has recently compiled a list of such functional Ψgs.

Depending on their mechanism of origin, Ψgs can be classified in three major classes: unitary, unprocessed and processed. Unitary Ψgs are derived from an ancestral protein-coding gene that has lost its protein-coding potential due to spontaneous mutations [11, 12]. While the unprocessed Ψgs originate from gene duplications that accumulate mutations, preventing their translation. On the other hand, processed pseudogenes (PΨgs), arise from the reverse transcription (retrotransposition) and integration of a processed mRNA into a new genomic location [13]. PΨgs lack the 5′ promoter sequence as well as any introns, however, they exhibit a 3′ polyA tail and duplications of varying length at its insertion site [14]. Recently, a new group of PΨgs have been identified in human and mouse, which lack the 3′ end poly-A tail and are derived by retrotranscription of circular RNAs (circRNA) [15].

PΨgs, are the most abundant type of Ψgs in the human genome with an estimated amount between ~ 8000 and 14,112 [11, 16–18]. As for today, GENCODE [12], the reference annotation for the human and mouse genomes, has annotated 10,822 human PΨgs (Release 37, GRCh38.p13) [19].

### Processed pseudogenes and cancer

Next-generation sequencing has contributed to the discovery of a large number of PΨgs and further studies have confirmed their involvement in the development, progression and prognosis of certain diseases, including cancer. A comprehensive list of PΨgs participating in the pathogenesis of different diseases has been compiled by Chen et al. [20]. Moreover, the detection of the transcribed PΨgs has demonstrated that certain PΨgs are expressed only in cancer samples, either in a specific cancer or in multiple cancers [21, 22]. For example, the *ATP8A2*-PΨg has been restricted to breast tumors with luminal histology showing a potential oncogenic nature [21]. In lung adenocarcinoma, the *PTPN12*-PΨg induces the removal of the *MGA* promoter, a likely tumor suppressor gene [23]. In gastric cancer, *POU5F1B,* a PΨg adjacent to *MYC*, is a prognostic marker [24], while in prostate cancer, the fusion of the *KLKP1*-PΨg and *KJK4* gene may be a potential biomarker in routine screening [25, 26].

Several PΨg integrations have been also identified, however no clear function nor correlation to disease has been yet understood. For instance, the *SMAD4*-PΨg is a confounding element in quantitative results and increases erroneous variant calls. Besides, the integration of the *SMAD4*-PΨg in the *SCAI* gene has been corroborated in hereditary cancer-predisposition cases [27], and while *SCAI* is characterized to have suppressive effect on tumor cell invasiveness, it has not been determined whether *SCAI* expression is hindered by the *SMAD4*-PΨg [28].

### Detection of processed pseudogenes

Ψgs were often discovered as by-product of gene sequencing or PCR experiments. With the advent of whole genome sequencing projects, computational approaches have aided in their identification and annotation, relying on the specific features of the Ψgs, such as level of sequence homology and completeness relative to a parent gene, lack of introns, ratio of non-synonymous to synonymous substitution rates (K_A_/K_S_), occurrence of polyadenine tail and the existence of frame disruptions, among others [29]. In eukaryotic genomes, some methods rely on homology-based approaches and these include in-house pipelines within genome-wide surveys [16, 30] and tools such as PseudoPipe [31], retroFinder [17] and PPFINDER [32], which unfortunately are not publicly available or are based on deprecated tools. Another type of algorithms relies on the information from mapped reads. The bioinformatics method developed by Cook et al. [23] detects somatically acquired Ψgs by aligning paired-end sequencing data to the genome and the transcriptome, nevertheless it is not publicly available. More recently, sideRETRO [33] was developed as a tool that focuses on the detection of de novo somatic and polymorphic insertions of PΨgs using a reference genome as well as a reference for the transcriptome. It applies a density-based clustering non-parametric algorithm and compiles the results in VCF format.

Today it is common that sequencing data analyses are performed by tech savvy staff, resulting in the use of formats that are burdensome to handle by researchers with basic computational skills. To aid in the prediction and interpretation of novel PΨg candidates, we present PΨFinder (P-psy-finder), a bioinformatics pipeline that rapidly screens alignments of DNA sequencing data to detect such events. It creates a simple table that can be sorted and filtered in any spreadsheet program, as well as graphical representations that besides providing a visual confirmation of the candidates, can be used to guide the experimental validation and the characterization of the genomic arrangement of such candidates. In addition, PΨFinder also provides information about known PΨgs found in the analyzed samples and can be used with any organism from whose genome is available.

## Implementation

### PΨFinder overview

PΨFinder aims to detect PΨgs within DNA sequencing data and predict their insertion sites. PΨfinder is written in python (3.6) [34] and requires STAR (2.7.7a) [35], SAMtools [36] (1.11), BEDTools [37] (v2.30.0) and R [38] (4.0.3).

**Fig. 1:**
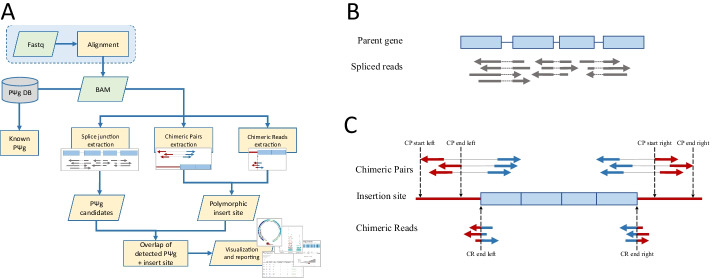
PΨFinder workflow. **A** Overall workflow of PΨFinder. If the input data are fastq files, these will be aligned using STAR. The resulting BAM file (input as default) is annotated with known PΨgs. Spliced reads, chimeric pairs and chimeric reads are extracted and combined to detect novel PΨgs and predict its insertion site. Results are displayed as text and html files. Different plots may be generated, including circular and linear layouts, scatter plots and summary dotplots. **B** Detection of a PΨg. Blue rectangles depict coding regions. Reads are depicted as arrows. Gray arrows refer to spliced reads, with dotted lines showing the splicing event. **C** Detection of the PΨg-insertion site. Blue arrows show reads mapping to the PΨg or portion of the PΨg, while red arrows show reads mapping to the PΨg insertion site or portion of the PΨg insertion site. Solid gray lines deem two reads as a pair. PΨFinder reports positions marked with dashed arrows. *CR* chimeric read, *CP* chimeric pair

The overall workflow is shown in Fig. 1A. For a given organism, PΨFinder takes fastq files as input and aligns them to the corresponding reference genome using STAR, a splice-aware aligner [35], alternatively alignment files can be supplied as input. To provide evidence of PΨgs in the sample, spliced reads (SR) across known exon-exon junctions are selected and clustered (Fig. 1B). To identify the insertion sites of the PΨg candidates, the pipeline extracts two pieces of information from the alignment files: (1) chimeric read pairs (CPs), pairs that are aligned in different chromosomes or at larger distances than expected, and (2) chimeric reads (CRs), soft-clipped reads (reads that align to two different locations) (Fig. 1C). The overlap between the PΨg candidates, CPs and CRs determine the PΨg’s insertion site. As output, PΨFinder provides summary reports in text and html formats as well as visualization of the predicted insertion sites (Additional file 1). Individual PΨgs and their insertion sites can be plotted either in linear or circular format (Fig. 1A). As a complementary result, PΨFinder also provides a list of detected known PΨgs.

## Results

### Screening for PΨgs in blood samples using PΨFinder

To demonstrate the use of PΨFinder, we scanned DNA sequencing data from 218 human blood samples. These samples were initially sequenced and analyzed (data not shown) using a custom-designed panel that included genes associated with hereditary colorectal cancer (Additional file 2, Additional file 3: Table S1). The comprehensive panel covers 28 genes and their promoter regions [39]. As a complementary analysis, these samples were scanned with PΨFinder using the human genome (hg19) as reference.

We detected a total of 423 PΨgs distributed across 209 samples. The predictions included PΨgs of only seven parent genes: *BMPR1A*, *CBX3*, *DHFR*, *HNRNPC*, *POLE*, *PTEN* and *SMAD4* (Table 1). PΨFinder detected only one PΨg in 34% of the positive samples, while 2 or more PΨgs were predicted in the rest of them (Additional file 3: Table S2). In terms of their genomic insertion site, the majority of the PΨgs were found either in intronic (54%) or intergenic (45%) regions, while only 1% had evidence of being inserted within an exon (*BMPR1A*-PΨg in 6 samples and *PTEN*-PΨg in 2 samples). The most common PΨgs detected were *CBX3*-PΨg and *BMPR1A*-PΨg, found in 180 and 155 samples, respectively. *PTEN*-PΨg and *DHFR*-PΨg were detected in 51 and 29 samples, respectively, while 6 samples contained *HNRNPC*-PΨg. *POLE*-PΨg and *SMAD4*-PΨg were found in one sample each.

**Table 1:** Summary of identified processed pseudogene candidates across all samples analyzed

Predicted PΨg	Number of samples predicted to harbor the PΨg (*N* = *218*)	Number of predicted PΨg-insertion sites, according to its insert site location^a^								
		Exonic			Intronic			Intergenic		
		CP-CR^b^	CP	CR	CP-CR	CP	CR	CP-CR	CP	CR
CBX3^c^	180 (82.6%)	0	0	0	123	3	59	0	0	0
BMPR1A	155 (71.1%)	0	5	1	0	321	99	1	320	134
PTEN	51 (23.4%)	0	1	1	0	131	27	0	151	39
DHFR	29 (13.3%)	0	0	0	0	39	8	0	17	6
HNRNPC	6 (2.8%)	0	0	0	0	0	0	3	1	2
POLE	1 (0.5%)	0	0	0	0	2	0	0	1	1
SMAD4^c^	1 (0.5%)	0	0	0	1	0	0	0	1	0

^a^Note that the sum of the predicted PΨg-insertion sites across different regions may vary from the total amount of samples, since one predicted PΨg may be predicted to have several insertion sites

^b^CP and CR stand for Chimeric Pair and Chimeric Read respectively. CP–CR denotes evidence from both Chimeric Pairs and Chimeric Reads supporting the insertion site, while the columns CP and CR denote only one type of chimeric evidence

^c^Selected PΨgs for experimental validation

The detection of the insertion sites of the novel PΨgs, relies on CPs and CRs, and while having both as evidence is not essential (one suffices to narrow down the insertion region), nevertheless, they do strengthen the accuracy of the predicted insertion site (Additional file 3: Table S4). In most of the insertion sites detected, only CPs (66.3%) or CRs (25.2%) gave supporting evidence, while a small percentage (8.5%) had support from both CPs and CRs. Among these well supported insertion sites we found (1) *CBX3-*PΨg located within the second intron of *C15ORF57* (chr15:40854180–40854180, in 119 samples), (2) *HNRNPC-*PΨg detected in the intergenic region between *LINC02541* and *MARCKS* (chr6:114017523–114017528, in 3 samples), (3) *BMPR1A-*PΨg inserted in the intergenic region between *PCGF5* and *HECTD2* (chr10:93083258–93083258, in 1 sample) and (4) *SMAD4-*PΨg located within intron 18 of *SCAI* (chr9:127732713–127732715, in 1 sample).

To validate these results, we selected PΨgs that besides having evidence from both CPs and CRs, they were inserted within an exon or an intronic region. This could provide evidence of a disease-causing mutation if the coding region of the disturbed gene were altered. *CBX3*-PΨg and *SMAD4*-PΨg complied with these criteria and were selected to experimentally determine their insertions sites using Sanger sequencing (Additional file 2).

### The ubiquitous CBX3-PΨg is reversed inserted in second intron of C15ORF57

**Fig. 2:**
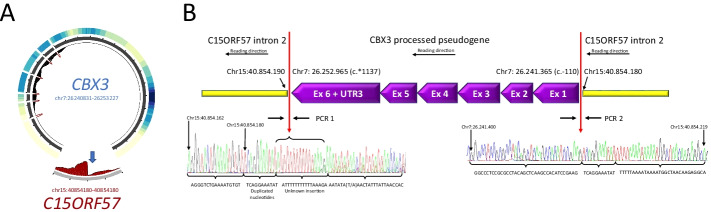
Experimental validation of *CBX3* processed pseudogene identified with PΨFinder. **A** Circos plot showing the coverage over *CBX3* and its insertion site in *C15ORF57* for sample113. The outer heatmap displays the coverage over the entire *CBX3* parent gene (only exons are depicted), darker color indicates higher coverage. The inner histogram shows the coverage over the exon-exon junctions, suggesting the presence of a PΨg. The red outmost histogram displays the coverage across the predicted insertion site in *SCAI*, shown by the arrow. **B** DNA sequence over the breakpoints of *CBX3-*PΨg inserted in intron 2 of the *C15ORF57* gene. Genomic coordinates refer to the human reference genome build hg19. Red arrows point out the actual breakpoints. Sequence for breakpoint 1 (PCR 1) includes an unknown insertion (ATTTTTTTTTTTAAAGA) and duplicated nucleotides (TCAGGAAATAT). Sequence for breakpoint 2 (PCR2) without any aberrations. *CBX3-*PΨg is reversed inserted on the minus strand, its parent gene is read from the plus strand, in the same reading orientation than the *CBX3* gene (see Additional file 2 for further details)

From RNA-seq data of lymphoblast tissue, *CBX3* has shown evidence to be expressed as a chimera with *C15ORF57* [40, 41]. This chimera has also been detected in multiple non-diseased tissues (tonsils, placenta, liver, skeletal muscle, adrenal gland and skin) from the Genotype Tissue Expression (GTEx) dataset [42] as well as in hepatocellular carcinoma [41] and glioblastoma [43]. In this study we present the DNA breakpoints of *CBX3* and *C15ORF57* as predicted with PΨFinder (Fig. 2A). The experimental validation showed an unknown insertion (ATTTTTTTTTTTAAAGA) and duplicated nucleotides (TCAGGAAATAT) in one of the breakpoints, while no aberrations were seen in the other breakpoint. *CBX3*-PΨg was found to be reversed inserted, aligning to the same reading orientation as *C15ORF57*. This makes the transcription of this fusion gene possible (Fig. 2B). The fact that *CBX3-*PΨg is recurrent (found in 82.6% of the samples) may suggest that it might have an effect in the predisposition to colorectal cancer development. However, in previous studies the *CBX3*-*C15ORF57* fusion was not only found in cancerous tissues, but also in normal or noncancerous samples [41, 42]. Although, experimental validations are needed, for example silencing the fusion through decreased cell proliferation and cell motility in specific cell populations, one might suggest that the expression level of this fusion gene might have an impact in cancer development.

### The well-known insertion site of the SMAD4-PΨg within the last intron of SCAI

**Fig. 3:**
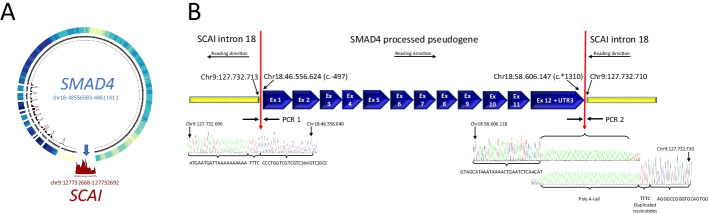
Experimental validation of *SMAD4* processed pseudogene identified with PΨFinder. **A** Circos plot showing the coverage over *SMAD4* and its insertion site in *SCAI* for sample220. The outer heatmap displays the coverage over the entire *SMAD4* parent gene (only exons are depicted), darker color indicates higher coverage. The inner histogram shows the coverage over the exon-exon junctions, suggesting the presence of a PΨg. The red outmost histogram displays the coverage across the predicted insertion site in *SCAI*, shown by the arrow. **B** DNA sequence over the breakpoints of *SMAD4-*PΨg inserted in intron 18 of *SCAI* gene. Genomic coordinates refer to human reference genome build hg19. Red arrows point out the actual breakpoints. Sequence for breakpoint 1 (PCR 1) includes a deletion of three nucleotides (GTC). Sequence for breakpoint 2 (PCR2) includes a poly A-tail and duplication of four nucleotides (TTTC). The *SMAD4-*PΨg is inserted in the minus strand in the same reading orientation as its parent gene in the opposite reading orientation than the *SCAI* gene (see Additional file 2 for further details)

Deleterious mutations in *SMAD4* have been shown to result in pancreatic cancer [44], juvenile polyposis syndrome [45], hereditary hemorrhagic telangiectasia syndrome [46] and Myhre syndrome [47]. The presence of the *SMAD4-*PΨg has interfered with diagnostic analyses based on clinical sequencing applications, creating false-positives results in 0.24–0.26% of the cases [27, 28]. Thus, its identification is crucial to reduce this confounding effect. We validated the breakpoints of *SMAD4* and *SCAI* as predicted with PΨFinder (Fig. 3A). In one of the breakpoints, we identified a deletion of three nucleotides (GTC), while in the other breakpoint a polyA-tail and a duplication of four nucleotides (TTTC) were confirmed [28] (Fig. 3B). The *SCAI* gene, might therefore lead to an upregulation of downstream genes involved in cancer development. The impact on the effect of the integration of *SMAD4* on the disruption of the function of *SCAI* needs to be further experimentally evaluated.

### Detection level and accuracy

**Fig. 4:**
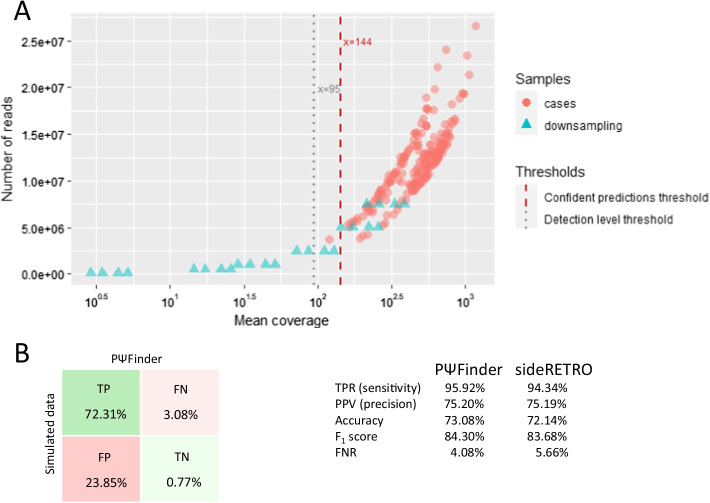
Detection level and Performance evaluation of PΨFinder. **A** Detection level based on sequencing depth. Samples with an average coverage of 144X or more (gray dashed line) are confident PΨg-insertion site predictions as determined by downsampling analysis (blue triangles, Additional file 3: Table S4). Samples with an average coverage of 95 or less (red dashed line) are below the detection level of PΨFinder. Samples between these thresholds are likely to be true predictions and manual inspection of the results is required. Biological samples are shown as red circles. **B** Performance evaluation. A total of 117 samples were analyzed. TP, true positives; TN, true negatives; FP, false positives; FN, false negatives; F_1_ score, harmonic mean of the precision and sensitivity; FDR, false discovery rate; TPR, true positive rate; PPV, positive prediction value

Sequencing depth plays an important role in any kind of computational predictions. To establish the detection level of PΨFinder, the four samples used for experimental validation were downsampled at 0.1, 0.5, 1.0, 2.5, 5.0 and 7.0 M paired reads, using seqtk (1.0) [48] (Additional file 3: Table S3). The resulting analysis with PΨFinder, determined that predictions obtained from samples with a sequencing depth of 5 M reads, an average coverage of at least 144X and including both CPs and CRs, can be deemed as true positive PΨg-insertion sites (Additional file 3: Table S4). Insertion site predictions based on samples with 2.5 M reads and an average coverage between 72 and 128X, start to lose evidence from either CPs or CRs. Thus, at this sequencing depth or “gray-zone” area, we recommend to further inspect the resulting predictions. Samples with 1 M sequencing reads or less and an average coverage of less than 52X over the panel, are outside the detection level of PΨFinder. Although we cannot entirely dismiss these predictions, they should be treated with caution. From the 218 colorectal cancer samples analyzed in this work, only one sample is within the “gray zone” with an average coverage of 120.71X (Additional file 3: Table S1), all others lie above the confidence prediction level (Fig. 4A). Considering this and the positive experimental validations all PΨgs detected that have supporting evidence from both, CPs and CRs, are most likely to be true.

To investigate the overall performance of PΨFinder we simulated 117 samples, each with a different PΨg inserted in a random position within the genome (Additional file 3: Table S5). An in-house script based on wgsim (0.3.0) [49] was developed defining the simulated error rate (2%) of the sequencing reads as well as their outer distance (500) and read length (90 bp). All samples contained 5 M simulated reads, yielding to a 98.8% of mapped reads with a mean coverage of 473X. These samples were analyzed with PΨFinder and sideRETRO (both with default values). The performance of both tools is remarkably similar (Fig. 4B) and their running time, while analyzing the simulated data, was in average one and two minutes per sample, respectively. Nevertheless, an advantage of using PΨFinder is the graphical visualization that it produces (Additional file 1) which aids in the confirmation of the predictions as well as in their experimental validation (Figs. 2, 3). Although the use of standard formats must be encouraged, e.g. sideRETRO using VCF files to report their results, these formats are still not easy to examine by researchers with basic computational skills, thus the html report and simple tabular format that PΨFinder generates is more convenient and user-friendly. Moreover, our tool reports known PΨgs that are found during the analysis.

## Conclusions

PΨFinder is a tool that can detect novel PΨgs from DNA sequencing data and determine their location in the genome. Here we demonstrated its application by scanning 218 DNA blood samples, from patients suspected of an inherited form of colon cancer, and identified 423 PΨgs from seven parent genes.

Among the predicted PΨgs, we identified the ubiquitous *CBX3-*PΨg, which has been shown to form a chimeric transcript with *C15ORF57* [40] and has been associated to glioblastoma [43] and hepatocellular cancer [41]. We validated its insertion site within intron 18 of *C15ORF57* and showed that *CBX3-*PΨg is reversed inserted. Although further expression and functional analyses are required, it may be likely that *CBX3-C15ORF57* could also be involved in the development of colorectal cancer.

We also detected *SMAD4* and validated its insertion site within the second intron of *SCAI*. *SMAD4*-PΨg is a known confounding element in the mutation analysis of next generation sequencing data in patients with juvenile polyposis syndrome or combined/juvenile polyposis/hereditary hemorrhagic telangiectasia [27]. Thus, it is important its identification to determine its relevance.

PΨFinder is a tool whose comprehensive and user-friendly results, can aid in the identification of PΨgs and complement any mutational screening in the identification of occurring mutations during cancer development and other diseases.

### Availability and requirements

Project name: Novel processed pseudogenes detection tool.

Project home page: https://github.com/bcfgothenburg/SSF.

Operating system(s): Linux, Mac OS.

Programming language: Python (3.6), bash.

Other requirements: STAR (2.7.7a), SAMtools (1.11), BEDTools (v2.30.0), R (4.0.3).

License: GNU General Public License, version 3.0 (GPLv3).

Any restrictions to use by non-academics: None (except the ones stated in GPLv3).

## Supplementary Information

**Additional file 1: Fig. S1.** PΨFinder summary report and visualization aids.

**Additional file 2**. Supplementary methods, experimental validation of *SMAD4-SCAI* and *CBX3-C15ORF57* including primers and gel pictures.

**Additional file 3: Tables S1–S5.** Sequencing data summary statistics and output from PΨFinder of case samples, downsampled data and simulated data. Benchmarking results.

## Data Availability

The code for PΨFinder, the user guide and test dataset are available in GitHub (https://github.com/bcfgothenburg/SSF). The datasets analyzed during the current study are available from the corresponding author on reasonable request.

## Abbreviations

PΨgs: Processed pseudogenes
PΨFinder: P-psy-Finder
circRNA: Circular RNAs
K_A_/K_S_: Ratio of the non-synonymous to synonymous substitution rates
HAVANA: Human and Vertebrate Analysis and Annotation
SR: Spliced read
CP: Chimeric pairs
CR: Chimeric reads
