# Supplementary material for: PΨFinder: a practical tool for the identification and visualization of novel pseudogenes in DNA sequencing data
Source: BMC Bioinformatics. 2022 Feb 3;23:59. doi: 10.1186/s12859-022-04583-4 (PMC8812246; doi:10.1186/s12859-022-04583-4)
Supplement: Supplementary file 1 — Additional file 1: Fig. S1. PΨFinder summary report and visualization aids. [file 12859_2022_4583_MOESM1_ESM.docx]

**Additional file 1. Supplementary Figure S1**

**PΨFinder: A practical tool for the identification and visualization of novel pseudogenes in DNA sequencing data**

Sanna Abrahamsson^1,†^, Frida Eiengård^2,†^, Anna Rohlin^2,3^ and Marcela Dávila López^1,*^

^1^ Bioinformatics Core Facility, Sahlgrenska Academy, University of Gothenburg, Sweden.

^2^ Department of Laboratory Medicine, Institute of Biomedicine, Sahlgrenska Academy, University of Gothenburg, Sweden.

^3^ Unit of Genetic Analysis and Bioinformatics, Department of Clinical Genetics and Genomics, Sahlgrenska University Hospital, Gothenburg, Sweden.

**
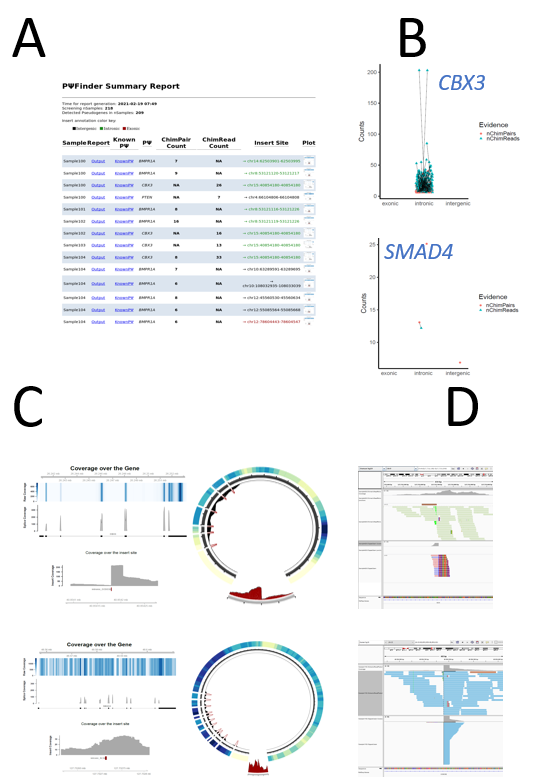
**

**Figure S1. PΨFinder summary report and visualization aids**. **A)** Summary report in html format, including all samples analyzed. Summaries are also provided in text format. **B)** Visualization plots of *CBX3* and *SMAD4***-**PΨgs. Scatter plots show the number of chimeric reads (blue) and chimeric pairs (red) supporting the detected PΨg in all samples analyzed. These are categorized as exonic, intergenic and intronic depending on where the insertion size was predicted. Lines between chimeric reads and chimeric pairs indicate both pieces of evidence belong to the same PΨg insertion site. **C)** Linear (default) and circular visualizations of the PΨg in one specific sample. **D)** IGV screenshots of the predicted insertion sites in one specific sample.
