# Supplementary material for: PΨFinder: a practical tool for the identification and visualization of novel pseudogenes in DNA sequencing data
Source: BMC Bioinformatics. 2022 Feb 3;23:59. doi: 10.1186/s12859-022-04583-4 (PMC8812246; doi:10.1186/s12859-022-04583-4)
Supplement: Supplementary file 2 — Additional file 2. Supplementary methods, experimental validation of SMAD4-SCAI and CBX3-C15ORF57 including primers and gel pictures. [file 12859_2022_4583_MOESM2_ESM.docx]

**Additional file 2. Supplementary Methods**

**PΨFinder: A practical tool for the identification and visualization of novel pseudogenes in DNA sequencing data**

Sanna Abrahamsson^1,†^, Frida Eiengård^2,†^, Anna Rohlin^2,3^ and Marcela Dávila López^1,*^

^1^ Bioinformatics Core Facility, Sahlgrenska Academy, University of Gothenburg, Sweden.

^2^ Department of Laboratory Medicine, Institute of Biomedicine, Sahlgrenska Academy, University of Gothenburg, Sweden.

^3^ Unit of Genetic Analysis and Bioinformatics, Department of Clinical Genetics and Genomics, Sahlgrenska University Hospital, Gothenburg, Sweden.

***Patients***

Patients were referred to the department of Clinical Genetics and Genomics at the University hospital (Gothenburg, Sweden) for diagnostic testing for hereditary colorectal cancer-predisposition genes. Genomic DNA was isolated from whole blood by using the BioRobot EZ1 (Qiagen, Hilden, Germany) with the EZ1 DNA Blood 350 ll kit (Qiagen). A custom-designed SureSelect hybridization enrichment assay (Agilent Technologies) containing genes connected to colorectal cancer was used and the comprehensive panel has previously been described in detail (1). The samples were sequenced using Illumina HiSeq 2000 (Illumina San diego, CA, USA) with 2x94 or 2x97 paired end reads resulting in fastq files with PE reads between 3,7M and 26,6M. Their mean coverage ranged between 120X and 1167X (Additional file 3: Table S1).

***Sanger Sequencing***

Primers were designed by using the tool Primer3 plus (http://www.bioinformatics.nl/cgi-bin/primer3plus/primer3plus.cgi) (Table 1). The products amplifications were performed on a 2720 Thermo cycler (Applied Biosystems) and were executed by a touchdown PCR (65-55)°C: 95°C 2 min, ((94°C 30 sec, 65°C 30 sec, 72°C 45 sec) 20 cycles, -0,5°C per cycle), (94°C 30 sec, 55°C 30 sec, 72°C 45 sec) 10 cycles, 72°C 7 min, 12°C ∞. Each PCR consisted of 12.9 µl of nuclease-free H_2_O, 4 µl of 5x MyTaq^TM^ Reaction Buffer (dNTP and Mg included), 1 µl of 10 µmol/L forward primer, 1 µl of 10 µmol/L reverse primer, 0.1 µl of MyTaq^TM^ polymerase and 1µl of approximately 75 ng/µl DNA (Bioline Reagents Ltd, United Kingdom). Detection and analysis of PCR products were made on E-gel EX 2% Agarose and E-Gel 48 2% Agarose (Invitrogen by Thermo Fischer Scientific) (Figure 1). Sanger sequencing was performed on 3500/3500xl (Applied Biosystems) and sequence chromatograms were analyzed with Sequence Analysis Software 6.0 (Applied Biosystems).

***Identification of the breakpoints of CBX3-C15ORF57***

To identify the exact insertion site of *CBX3*-PΨg within the 2^nd^ intron of *CBX3,* four primers were designed. The gene *CBX3* has its reading direction on the (+) strand while the gene *C15ORF57* (*CCDC32*) has its reading direction on the (–) strand. In our case, the integration of *CBX3*-PΨg has shown to be inverted and according to (2), this leads to a new fusion gene that starts with exon 1 and 2 of *C15ORF57* and ends with exon 1 to 6 of the *CBX3*-PΨg. One amplified amplicon, spanned over the 5´end of the *CBX3-*PΨg, comprising the *C15ORF57*-bound forward primer and the UTR3 region of *CBX3* reverse primer. The amplification was made according to description under *Sanger sequencing*. A second amplicon was made over the 3´end of the *CBX3*-PΨg with a forward primer located in *CBX3* exon 2 and the reverse primer located in *C15ORF57* (Figure 2B in main text)*.*

***Identification of the breakpoints of SMAD4-SCAI***

To validate the predicted insertion site of *SMAD4*-PΨg in the 18^th^ intron of *SCAI* four primers were designed. The first amplicon amplified, spanned over the 5´end of the *SMAD4*-PΨg, compromising the *SCAI*-bound forward primer and the *SMAD4* exon 3 reverse primer. The amplification was made according to the description under *Sanger Sequencing,* adding 1.6 µl DMSO to each PCR reaction due to the GC-rich area. The second amplicon was amplified over the 3´end of the *SMAD4-*PΨg, with a forward primer located in the UTR3 region of *SMAD4* and the reverse primer located in *SCAI* (Figure 3B in main text).

1. Rohlin A, Rambech E, Kvist A, Torngren T, Eiengard F, Lundstam U, et al. Expanding the genotype-phenotype spectrum in hereditary colorectal cancer by gene panel testing. Fam Cancer. 2017;16(2):195-203.

2. Zhu C, Wu L, Lv Y, Guan J, Bai X, Lin J, et al. The fusion landscape of hepatocellular carcinoma. Mol Oncol. 2019;13(5):1214-25.

**Table 1. Primers**

| *SMAD4 - SCAI* |  |
| --- | --- |
|  |  |
| PCR1 (breakpoint 1) |  |
| Primer | Sequence 5´- 3´ |
| Forward (SCAI F3) | AAACTTGGAAGCAACCCAAA |
| Reverse (SMAD4 ex 3R) | AGGAAATCCTTTCCGACCAG |
|  |  |
| PCR2 (breakpoint 2) |  |
| Primer | Sequence 5´- 3´ |
| Forward (SMAD4 UTR3) | TTGTATCTTGGGGCAAGACTG |
| Reverse (SCAI R1) | TGCAATGACTCGATCTCAGC |
|  |  |
| *CBX3 - C15ORF57 (CCDC32)* |  |
|  |  |
| PCR1 (breakpoint 1) | Sequence 5´- 3´ |
| Forward (ORF F2) | TATTCCCAGGTTTGGCTTTG |
| Reverse (CBX3 UTR F2) | AATGTTGGAATCCCTGTTGC |
|  |  |
| PCR2 (breakpoint 2) | Sequence 5´- 3´ |
| Forward (CBX3 ex2R) | GGACATTTCCCCCAACATTT |
| Reverse (ORF R1) | CCAGGGCTACATGTGGATTT |


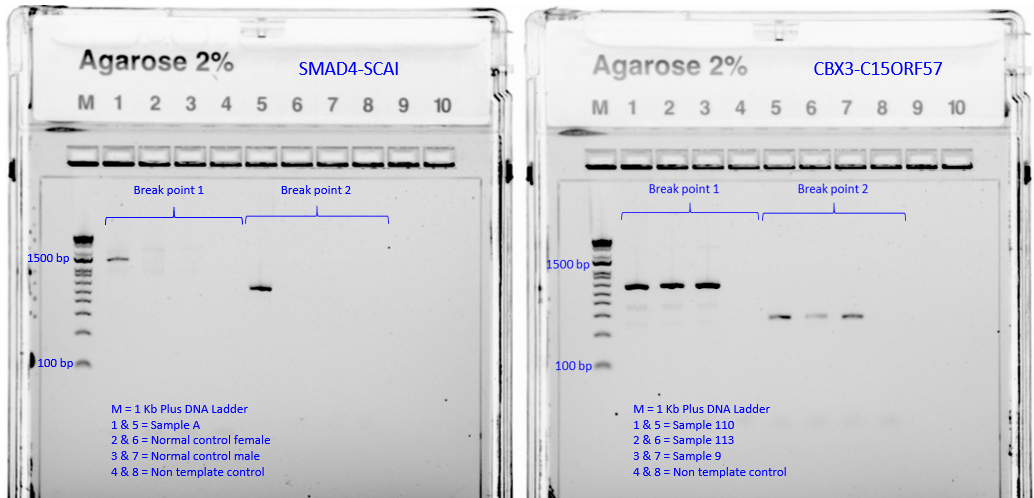


**Figure 1. Gel pictures of PCR products.** The picture on the left shows the PCR amplification over the breakpoints of the PΨg complex *SMAD4-SCAI.* The picture on the right shows the amplification over the breakpoints for PΨg complex *CBX3-C15ORF57*. Description of the content in the wells is described in the pictures. Primers were designed so only samples that contain the PΨg will result in one fragment in PCR amplification.
